# Intracellular and Extracellular Markers of Lethality in Osteogenesis Imperfecta: A Quantitative Proteomic Approach

**DOI:** 10.3390/ijms22010429

**Published:** 2021-01-04

**Authors:** Luca Bini, Domitille Schvartz, Chiara Carnemolla, Roberta Besio, Nadia Garibaldi, Jean-Charles Sanchez, Antonella Forlino, Laura Bianchi

**Affiliations:** 1 Functional Proteomics Laboratory, Department of Life Sciences, University of Siena, 53100 Siena, Italy; luca.bini@unisi.it (L.B.); c.carnemolla@futura-fm.com (C.C.); 2 Division of Laboratory Medicine, Department of Medicine, University Medical Center, 1206 Geneva, Switzerland; Domitille.Schvartz@unige.ch (D.S.); Jean-Charles.Sanchez@unige.ch (J.-C.S.); 3 Biochemistry Unit, Department of Molecular Medicine, University of Pavia, 27100 Pavia, Italy; roberta.besio@unipv.it (R.B.); nadia.garibaldi01@universitadipavia.it (N.G.); 4 Istituto Universitario di Studi Superiori-IUSS, 27100 Pavia, Italy

**Keywords:** osteogenesis imperfecta, extracellular matrix, cytoskeleton, cell signaling, bioinformatics, REVIGO, pathway analysis

## Abstract

Osteogenesis imperfecta (OI) is a heritable disorder that mainly affects the skeleton. The inheritance is mostly autosomal dominant and associated to mutations in one of the two genes, *COL1A1* and *COL1A2*, encoding for the type I collagen α chains. According to more than 1500 described mutation sites and to outcome spanning from very mild cases to perinatal-lethality, OI is characterized by a wide genotype/phenotype heterogeneity. In order to identify common affected molecular-pathways and disease biomarkers in OI probands with different mutations and lethal or surviving phenotypes, primary fibroblasts from dominant OI patients, carrying *COL1A1* or *COL1A2* defects, were investigated by applying a Tandem Mass Tag labeling-Liquid Chromatography-Tandem Mass Spectrometry (TMT LC-MS/MS) proteomics approach and bioinformatic tools for comparative protein-abundance profiling. While no difference in α1 or α2 abundance was detected among lethal (type II) and not-lethal (type III) OI patients, 17 proteins, with key effects on matrix structure and organization, cell signaling, and cell and tissue development and differentiation, were significantly different between type II and type III OI patients. Among them, some non–collagenous extracellular matrix (ECM) proteins (e.g., decorin and fibrillin-1) and proteins modulating cytoskeleton (e.g., nestin and palladin) directly correlate to the severity of the disease. Their defective presence may define proband-failure in balancing aberrances related to mutant collagen.

## 1. Introduction

Osteogenesis imperfecta (OI) is a clinically and genetically heterogeneous rare disease (1/10,000–15,000 live births) affecting mainly bone, but impairing also extraskeletal tissues functionality [1]. It is mainly inherited as an autosomal dominant disorder (80–85%) caused by different mutations in either *COL1A1* (Mendelian Inheritance in Man, MIM #120150) or *COL1A2* (MIM #120160) genes, which encode the α1 and α2 chains of type I procollagen, respectively.

*COL1A1*/*COL1A2* mutations may cause quantitative (haploinsufficiency) or qualitative defects of the collagen type I. Haploinsufficiency determines the milder form of the disease (MIM #166200) with reduced presence of structurally normal collagen [2]. Conversely, dominant qualitative defects, causing chain sequence alteration and synthesis and secretion of abnormal procollagen molecules, result in a wide spectrum of phenotypes: from perinatal lethal type II to moderate type IV based on OI Sillence classification (OI type II: MIM #166210; OI type III: MIM #259420; OI type IV: MIM #166220) [2].

Although the mutant collagen is generally secreted by the cells and responsible for the assembly of an abnormal extracellular matrix (ECM), a fraction of the protein is retained in the endoplasmic reticulum (ER), causing cellular stress responsible for unfolded protein response, autophagy, and apoptosis activation.

Mutations in *COL1A1* are typically more severe, and frequent, than those in *COL1A2* [2,3]. About 80% of *COL1A1*-related OI are determined by a glycine substitution in one of the highly conserved Gly-X-Y tripeptide in the triple helical domain. About one-third of these substitutions results in a lethal phenotype [4]. In contrast, less than 20% of these type of substitutions in the α2 (I) chain causes lethal outcome [4].

The genotype-phenotype correlation is still debated in OI [2,4,5,6] and no definitive agreement exists on why both different mutations in collagen I chains as well as identical mutations in *COL1A1*/*COL1A2* may have very different OI clinical manifestations [2,5,7].

In the OI murine model Brtl ^+/−^, which develops both lethal and non-lethal outcomes in presence of the same α1 (I)-Gly349Cys mutation, a different response of bone cells to the presence of mutant collagen was reported in mice with a different phenotype [8]. Furthermore, Bianchi et al. described an abnormal cytoskeleton organization compromising osteoblast proliferation, collagen deposition, integrin, and TGF-β signaling, and related impairment of bone structural properties in lethal and non–lethal Brtl ^+/−^ mice [9]. Aberrant cytoskeleton assembly was also confirmed in fibroblasts from lethal OI patients carrying identical Gly substitutions in α1 (I) [9]. More recently, cytoskeleton and nucleoskeleton defects in severe/lethal OI recessive forms (OI type VII: MIM #610682; OI type VIII: MIM #610915; OI type IX: MIM #259440) have been also highlighted (these OI type numbers refer to the online Catalog of Human Genes and Genetic Disorders, OMIM) [10].

Here, to advance in defining which differentially affected pathways reflect or correlate to different patient capability in coping with the disease and to understand how different modulation of those pathways may account for phenotype heterogeneity, we used human fibroblast samples from patients carrying mutations in *COL1A1* or *COL1A2* genes, with both lethal and non-lethal phenotypes. Although the small number of patients used for the study does not allow us to make any definitive conclusion, performing protein quantification by quantitative tandem mass tags (TMT) isobaric labeling and REVIGO and MetaCore data processing, we identified a particular fibroblast protein profile differentiating type III from type II OI probands, regardless of the causative mutation and the affected *COL1A1* or *COLA2* gene.

## 2. Results and Discussion

Osteogenesis imperfecta is a generalized connective tissue disorder with a wide spectrum of mutations and phenotype manifestations. No definitive therapy currently exists for this disease [11] and the comprehension of cellular and biochemical mechanisms that determine OI molecular aberrances is crucial in the attempt to improve its clinical management and innovative and more effective therapies development. En route for this ambitious goal, we attempted to ameliorate the understanding of OI affected molecular pathways and of their possible influence on the outcome of the disorder by identifying protein differences occurring among lethal OI type II and non-lethal OI type III patients, which carry glycine substitution at different positions along the α chains of collagen type I.

Primary fibroblasts from dominant OI probands, three carrying mutations in the *COL1A1* gene: two with lethal and one with severe outcome, and three with mutations in the *COL1A2* gene: one with lethal and two with severe outcome (Table 1), were analyzed by quantitative mass spectrometry (MS) using isobaric tandem mass tag (TMT) technology. We already previously proved that these lines do not show specific differences in unfolded protein response, autophagy, or apoptosis activation associated to the patient outcome severity [12]. To delineate how different capabilities in handling compromised cellular functions may modulate OI outcome, the identified protein patterns were processed to find out proteins present at different levels among lethal and survived subjects, independently on both causative mutated gene and position of the mutation. Seventeen proteins significantly differed in abundance between the lethal and the non-lethal patients (quantitative ratios above 1.5 or below 1/1.5, *p* ≤ 0.001), with about 2/3 of the proteins (*n* = 11) presenting increased levels in cells from lethal probands (Table 2). Not surprisingly [9], in the fibroblasts from survived patients, the 17 proteins were detected with an intermediate concentration of those of controls and lethal cells (Table 2). No difference in collagen amount was detected among the samples, suggesting that it was likely not responsible for the different outcome in the analyzed fibroblasts. This indicates that mutant collagen may not directly exert the main role in defining the lethal outcome of the disorder, as already proved for the OI murine model Brtl [13]. Rather, the capability in mutant collagen managing and in counterbalancing secondary aberrances, induced by its occurrence, seems crucial for the survival of patients enrolled in this study.

**Figure 1:**
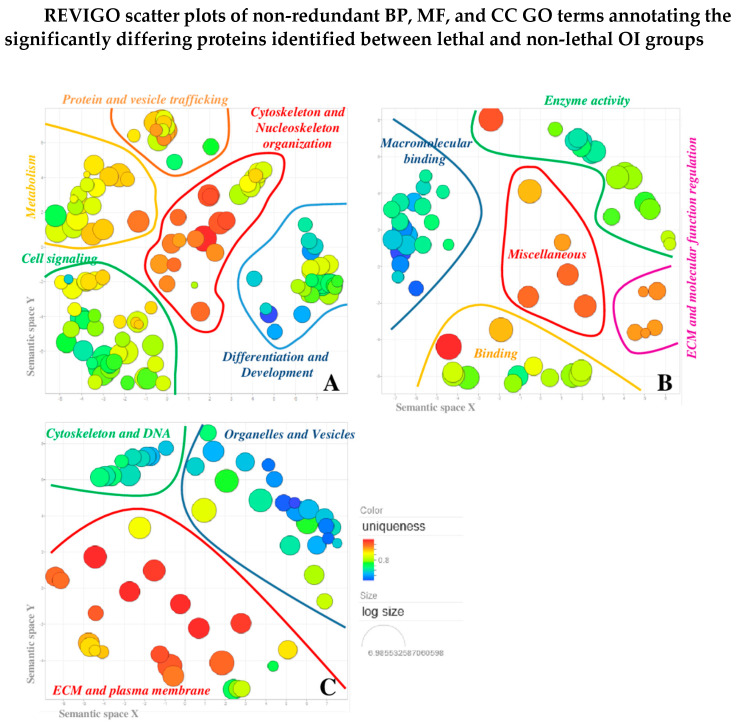
REVIGO scatter plots of non-redundant Gene Ontology (GO) terms annotating the 17 differentially abundant proteins identified between lethal and non-lethal OI groups. Individual plots were built for the three ontologies: biological process (**A**), molecular function (**B**), and cellular component (**C**). Each bubble represents a summarized GO term and the more semantically similar are closer in the plot. The main resulting clusters were graphically highlighted by curved lines. GO terms that were significantly over- or under-represented (GO annotation uniqueness) in the GO list were highlighted by bubble color: from brilliant red, for common shared terms, to dark blue for unique individual terms. Bubble size indicated the frequency of individual GO term in the GO annotation (GOA) referring database (SwissProt): larger the radius, higher in hierarchy is the corresponding GO term.

The biological meaning of the differentially quantified proteins was at first exploited by a general overview on Gene Ontology (GO) terms annotating the 17 proteins of interest in UniProtKB. Biological process (BP), molecular function (MF), and cellular component (CC) GO terms were summarized (i.e., redundancy reduction) and visualized by applying REVIGO resource, as shown in Figure 1A–C. In the REVIGO scatter plots, GO terms were clustered based on semantic similarities and irrespective of related proteins. Curved lines were drawn to graphically highlight the main resulting semantic-groups. For greater clarity, the GO terms summarized by REVIGO were listed around each scatterplot according to their distribution into the main delineated term groups (Supplementary Figure S1A–C).

The BP term REVIGO scatter plot provides the more functional information, whereas MF and CC term processing is extremely useful to corroborate the BP term analysis. In particular, they strengthened the hypothesis of a “domino effect” in OI molecular and biochemical affections that falls from ECM to nucleoskeleton, though cell membrane and cytoskeleton, and vice versa, as we previously described [9,10].

The BP REVIGO scatter plot (Figure 1A) highlighted five main clusters, in which BP GO terms were distributed by the software. Based on the meaning of the clustered terms, groups were named as follows: (i) *cell signaling*, (ii) *differentiation and development*, (iii) *cytoskeleton and nucleoskeleton organization*, (iv) *metabolism*, and (v) *protein and vesicle trafficking*.

The most significant result emerging from BP REVIGO analysis is the close functional interdependence existing between the cellular processes delineated by the BP built clusters, as the functionality of the one is cause and effect of the functionality of the other. In fact, if on one hand, signal transduction is cause and effect of protein and vesicle trafficking as well as of cytoskeleton and nucleoskeleton organization, on the other, cytoskeleton and nucleoskeleton regulate molecular trafficking, compartmentalization, organelle functions, gene expression, and metabolism. Indeed, cellular differentiation and tissue and organism development are the overall result of the complex functional framework defined by the molecular activities expressed in the other four clusters.

**Figure 2:**
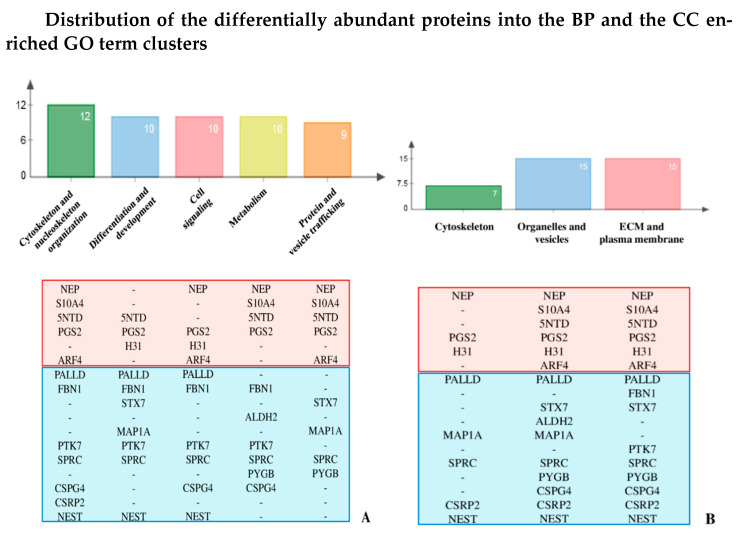
Distribution of the differentially abundant proteins into the BP (**A**) and the CC (**B**) enriched GO term clusters - enrichment analysis results were improved by literature text mining. The number reported in each bar indicates the proteins, whose acronyms are listed below histograms, included in the corresponding cluster. Proteins in the light-red area resulted down-regulated while those in the light-blue area were found up-regulated in lethal probands.

Moreover, several of the proteins of interest are annotated by GO terms grouped in different REVIGO clusters (Figure 2). They in fact localize in different cellular/extracellular microenvironments and are involved in distinct and interconnected cellular-functions, whose affection is supposed or partially described concurring to OI defects and severity.

The majority of BP terms, corresponding to 10 protein differences, belong to the *cell signaling* cluster, (i), thus suggesting that different levels in signaling may play a crucial role in defining the lethality or the survival outcome of the disorder. In addition, 7 out of the 10 proteins annotated by cluster (i) BP terms are also annotated by GO terms from the *differentiation and development cluster*, (ii). Based on (i) and (ii) GO term meaning, REVIGO analysis highlighted that defective response to extracellular *stimuli*, which are involved in key processes of differentiation/development and homeostasis, represents one of the possible OI molecular aberrances with negative impact on the ability of probands to counterbalance lethal effects of *COL1A1/COL1A2* mutations.

In line with numerous observations of extra-skeletal OI manifestations and with our previous investigations [1,9,10,14,15,16,17,18], BP GO terms from (ii) indicated a possible exacerbation of differentiation/development and homeostasis defects in bone, central nervous system, heart/vasa, ear, eye, lung, and skeletal muscle from lethal patients (Supplementary Figure S1, *differentiation and development* cluster).

**Figure 3:**
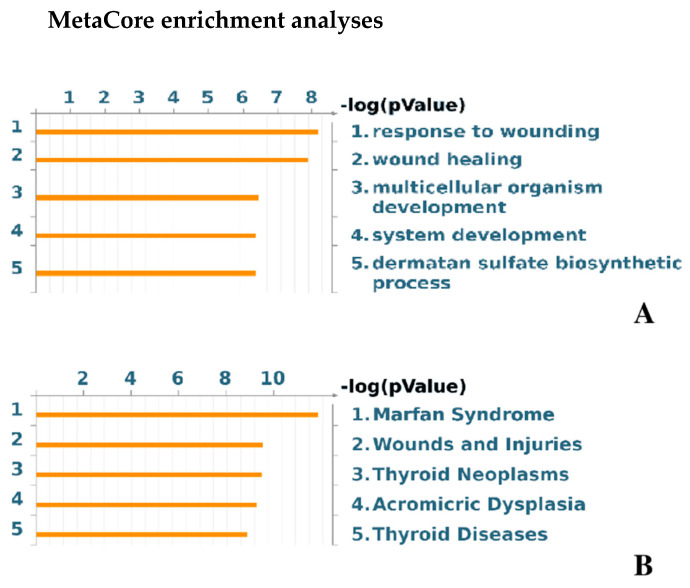
MetaCore enrichment analyses in the “disease by biomarkers” (**A**) and in the “GO processes” (**B**) ontologies.

The BP GO terms “sequestering of TGF-β in extracellular matrix” and “sequestering of BMP in extracellular matrix” emerged among the *cell signaling* annotations. They suggested an altered TGF-β and BMP bioavailability control in lethal probands. Since they trigger molecular cascades that induce, control, and modulate proliferation and differentiation in osteoblasts as well as in many other cell types, including fibroblasts, these multipotent cytokines have, depending on their different bioavailability, dramatic effects on target cells and thus a possible detrimental role in OI clinical outcome [19,20]. In particular, any factor interfering on TGF-β active and latent forms may impair bone formation, healing, and remodeling [18,21,22,23,24]. During osteoclast bone resorption, TGF-β is actually released by the ECM and its active form concurs to osteoblast progenitor recruitment and differentiation [25,26,27,28]. Conversely, elevated activation of this cytokine delays osteoblast differentiation and matrix mineralization [26]. According to MetaCore enrichment analysis in the “disease by biomarkers” ontology, defects in response to wounding, wound healing, and development are suggested as modulators of the OI severity (Figure 3A). Therefore, protein differences that are functionally described by the two above reported GO terms may have pivotal roles in determining the fate of the proband.

### 2.1. Fibrillin-1 Higher Abundance in Lethal Fibroblasts: Signaling and Metabolism as Possible Players for OI Phenotype

**Fibrillin-1** (FBN1) is a glycoprotein with structural function in the ECM that was found, in the present analysis, up-regulated in lethal-patients (Table 2). It was reported to modulate endogenous TGF-β and BMP bioavailability by inducing their sequestration in the ECM [28,29,30]. As suggested by our previous findings on bone samples from the OI murine model Brtl ^+/−^, TGF-β and BMP latent forms may accumulate in the ECM of lethal patients [18]. This could result in a reduction of TGF-β/BMP signaling during bone anabolism and in an excessive and uncontrolled active-form release of these cytokines during osteoclast bone-matrix resorption. Hence, TGF-β and BMPs aberrantly stored in ECM are a “time bomb” whose triggering, even by physiological bone remodeling, may lead to the osteoblast and matrix defects described in OI. In supporting the hypothesis of increased TGF-β signaling involvement in abnormal skeletal properties, OI mice are known to present improved bone mass and strength when treated with TGF-β inhibiting antibodies [31,32]. Reasonably, aberrant increased concentrations of fibrillin-1 in lethal patients may represent a negative modulator of OI phenotype by exacerbating TGF-β signaling consequences.

**Figure 4:**
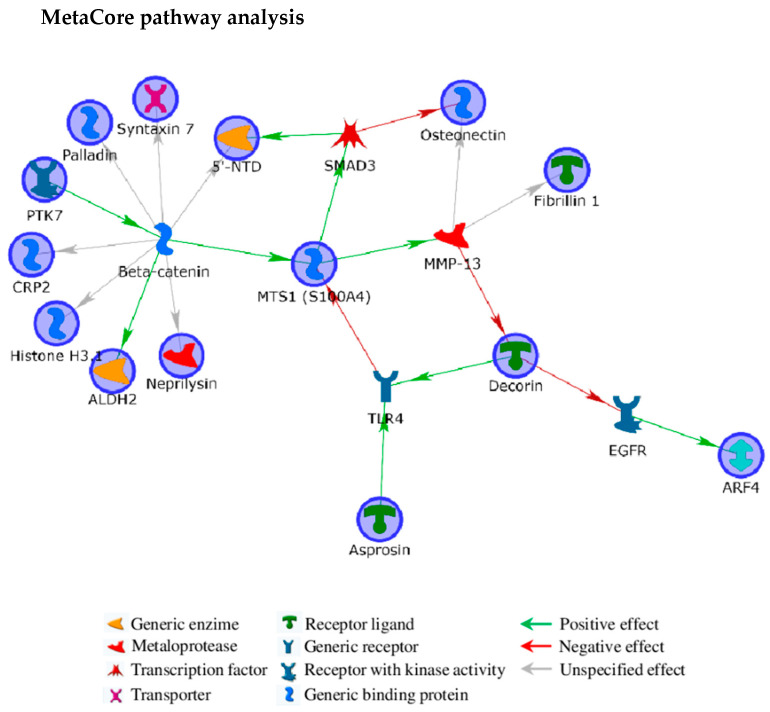
MetaCore protein network built by processing significant protein differences occurring among fibroblasts from dominant OI patients with lethal or non-lethal outcome. Experimental proteins, circled in blue, were cross-linked by using the shortest path algorithm. Only four of them did not enter the net (i.e., Microtubule-associated protein 1A; glycogen phosphorylase, brain form; chondroitin sulfate proteoglycan 4; nestin), thus suggesting a tight functional correlation among identified differences. Functions of network objects are visualized by different symbols, as reported in the legend. Edge colors and arrowheads indicate type and direction of protein interconnection, respectively.

Interestingly, secreted profibrillin generates fibrillin-1 and **asprosin** by furin cleavage. Asprosin is a protein hormone involved in glucose homeostasis regulation that is largely produced by white adipose tissue [30]. Alterations in energy metabolism were described in the *Col1a1^Jrt/+^* OI mouse model and hypothesized to contribute to OI pathophysiology [33]. Since asprosin is commonly produced by profibillin maturation, the increase of fibrillin-1 in lethal patients may be associated to an enhanced release of asprosin that could result in glucose metabolism defects. In type II OI fibroblasts, we also observed an up-regulation of the **mitochondrial aldehyde dehydrogenase** (ALDH2) and of the **glycogen phosphorylase** (PYGB) that may corroborate the hypothesis of glucose metabolism impairment in OI, as further stressed by the BP GO term *metabolism* cluster (Supplementary Figure S1A). Interestingly, the Wnt/β-catenin signaling, which is defective in OI [34], was described causing a shift from oxidative phosphorylation to glycolysis [35], as also suggested by the functional interaction between ALDH2 and β-catenin in the MetaCore network (Figure 4). Moreover, the hypothesized mechanistic coordination between ECM deposition/degradation and energy metabolism [36,37] further stresses the possible correlation between dysfunctions in glucose metabolism and ECM aberrances that our work suggests.

The relevance of fibrillin-1 abundance defects in OI severity intriguingly suggests a recall to the Marfan syndrome (MaS) (MIM #154700), another complex connective tissue disorder. Fibrillin-1 gene mutations are in fact causative of MaS which predominantly affects cardiovascular, respiratory, and ocular systems, together with abnormalities associated to the skeleton [38,39]. MetaCore enrichment analysis in the “diseases by biomarkers” ontology emphasizes OI correlation to Marfan syndrome and to acromicric dysplasia (ACMICD) (MIM #102370) (Figure 3B). This latter is a recently described autosomal dominant disorder characterized by severe short stature, short hands and feet, joint limitations, and skin thickening [40]. Interestingly, FBN1 mutations resulting in ACMICD localize in the exon region encoding the TGF-β-binding protein-like domain-5 [41].

Finally, fibrillin-1 directly interacts with the small dermatan sulfate proteoglycan decorin whose altered expression has been described in neonatal Marfan syndrome [42] and in ECM from OI patients [43].

### 2.2. Decorin Reduction May Modulate OI Severity Acting on Cell Signaling, Intracellular Trafficking, Cytoskeletal Organization, and Collagen Assembly

Decorin is another ECM structural component that our preliminary analysis showed with different concentrations in fibroblasts from dominant OI with lethal and non-lethal outcome. Its abundance is reduced in both OI samples compared to control and in lethal vs. non-lethal probands. Decorin is a pleiotropic and multifunctional protein playing crucial roles in all the five functional classes delineated by BP GO annotations (Figure 2, PGS2). As underlined by the BP GO term “cytokine mediated signaling pathway”, from the *cell signaling* cluster, decorin is known to control TGF-β bioavailability by direct interaction with this latter and by down-regulating its production [44,45].

Interestingly, some ECM components were suggested to control autophagic signaling pathways and decorin was reported to induce autophagy by interacting with the Epidermal Growth Factor Receptor (EGFR) [46,47]. ER procollagen aggregates, constituted by aberrant collagen type I molecules, are eliminated by autophagy [48,49]. Since it is a driving force in cell survival, autophagy may play a fundamental role in controlling ER stress in OI, thus bending the needle of the balance towards the survival of the probands. As EGFR signaling is known to be related to the **ADP-ribosylation factor 4** (ARF4) activation [50,51], decorin may also indirectly control molecule and vesicle trafficking through ARF4. Furthermore, ARF4 is involved in ECM protein deposition [36,37,52] and Wnt transport and exocytosis [53], indeed, decorin may influence, through EGFR and ARF4, ECM production and Wnt signaling. This tight cross talk among decorin, EGFR, and ARF4 is clearly evidenced by the MetaCore built network (Figure 4). Notably, while ARF4 resulted down-regulated in fibroblasts from type II OI patients, a significant increase of this protein was detected in type III OI probands (Table 2).

Defects in protein and vesicle trafficking correlated to Wnt/β-catenin, as highlighted by the MetaCore network, are further suggested by the consistent increased in lethal patients vs. controls (Table 2) of the **syntaxin-7 protein**, a Q-SNARE protein active in phagocytosis vesicle trafficking [54].

Besides Wnt/β-catenin, decorin was also reported to interfere with Myc and hypoxia inducible factor-1α (HIF-1α) signaling [55], they all suggested or proved to be affected in dominant and in some recessive OI forms [10,34].

Among its GO BP specifying terms, the “negative regulation of JAK-STAT cascade” annotation, from the *cell signaling* cluster, stressed another crucial role that decorin seems to have in osteoblast differentiation, bone development, and wound healing. JAK-STAT signaling was described as a negative modulator of mesenchymal stem cell osteogenic differentiation [56]. Decorin, operating as an inhibitor of JAK-STAT signaling [57], may hence support osteoblast differentiation during bone formation and remodeling.

As it modulates α_2_β_1_ integrin during matrix synthesis, decorin was furthermore described to influence cytoskeleton organization by controlling the vimentin intermediate filament system [58]. Decorin down-regulation is actually associated to vimentin fibrils reduction [58], with possible cytoskeleton disorganization and, in extreme cases, cytoskeleton collapse [59,60]. Being vimentin fundamental in focal adhesion assembly and stability, in cell and tissue mechanical strength and integrity, as well as in cytoskeleton organization and organelle distribution and function [60,61,62,63], decorin reduced abundance may affect cell adhesion and polarity, molecular trafficking and cell signaling, as well as cell and tissue wound healing processes, by even acting on vimentin. A possible involvement of vimentin decrease in lethal phenotype penetrance was already reported by Bianchi et al., 2012, that described lower levels of vimentin in mutant lethal Brtl ^+/−^mice [18].

Decorin regulates collagen I fibril shape, size, and organization in the ECM [39,64], as stressed by its GO BP term “extracellular matrix organization”, from the *cytoskeleton and nucleoskeleton organization* cluster. Its decrease in lethal cases may concur to impair ECM physicochemical and functional properties in OI patients. Indeed, decorin knock-out mice (Dcn ^−/−)^ present skin and tendon fragility, delayed wound healing, and, in association with biglycan knock out (Bgn ^−/−^), decreased osteogenesis, and osteoporosis-like phenotype [65,66,67,68]. Furthermore, the reduced binding of decorin to mutant collagen, which was reported in patients affected by recessive OI type VII (*crtap* ^−/−^) [31], stresses the decorin down-regulation as a negative cellular response in modulating OI severity. Of relevance, type I collagen mutations affecting proteoglycan binding sites are often perinatal lethal in OI [69].

Decorin aberrant presence may also contribute to another OI feature, as suggested by its annotating GO terms: “skeletal muscle tissue development” and “response to mechanical stimulus”, from *differentiation and development* and *cytoskeleton and nucleoskeleton organization* clusters, respectively. Given the interdependence of bone and skeletal muscle development and function, OI patients show an altered skeletal muscle physiology [70]. Many OI patients report intolerance to physical activity, fatigue, and muscle weakness, and, in some cases, these phenomena are so severe that they are valued as symptoms of the disease [16]. Despite it not being the main collagenic component in muscle ECM, mutant collagen type I is thought to have a detrimental effect also on muscle-ECM properties and function [70]. Indeed, OI muscle deficiency does not merely result from motor difficulties and inactivity; it likely depends also on the incapability of muscle connective tissue to respond to mechanical stimuli during contractile force transmission. Decorin is known to influence tissue tensile strength and to improve muscle mass by decreasing expression of myostatin and indirectly increasing the expression of dystrophin [71]. Consequently, its deficiency may concur to muscle weakness in osteogenesis imperfecta patients. Of great relevance, decorin was suggested for a combined therapy in Duchenne muscular dystrophy [71].

**Figure 5:**
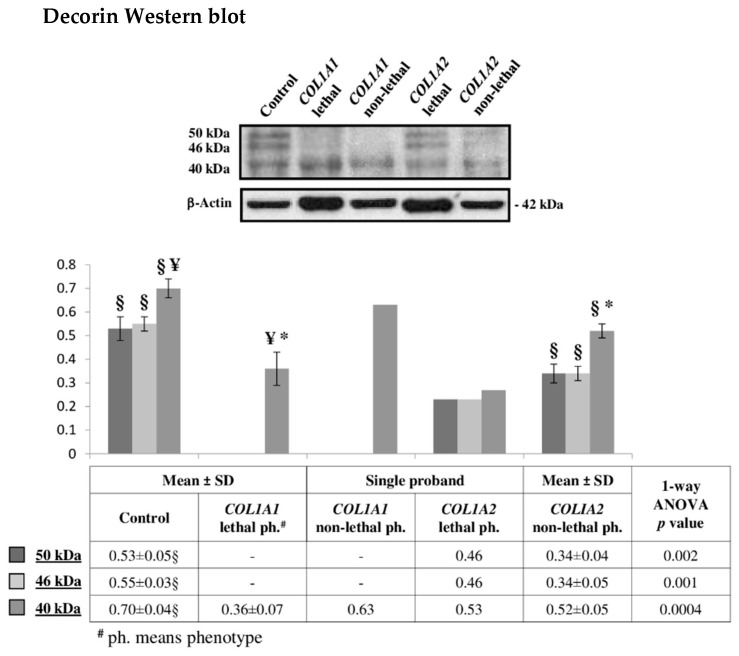
Representative decorin Western blot on fibroblasts from dominant OI patients carrying lethal or non-lethal mutations in *COL1A1* or in *COL1A2* genes. The shown signals of immunostained bands from controls, *COL1A1* lethal, and *COL1A2* non-lethal mutants are representative of the corresponding sample classes. Histograms visualize the mean of normalized relative-integrated-density ± SD values of controls, *COL1A1* lethal and *COL1A2* non-lethal subjects, and normalized relative-integrated-density for *COL1A1* non-lethal (*n* = 1) and *COL1A2* lethal (*n* = 1) fibroblasts. Statistics was performed on normalized mean relative-integrated-density ± SD values, which were computed on values of Western blot signals from controls (*n* = 3), lethal *COL1A1* mutants (*n* = 2), and non-lethal *COL1A2* mutants (*n* = 2). §, ¥, and * symbols indicate significant abundance changes occurring between controls and *COLIA2* non-lethal mutants, between controls and *COL1A1* lethal phenotype, and between *COL1A1* lethal and *COLIA2* non-lethal mutants, respectively.

Following up the above discussed critical functions performed by decorin in modulating several processes and structures affected in OI patients, we verified the differential occurrence of decorin in type II and type III OI patients by Western blot (Figure 5). The relative-integrated-density of the anti-decorin immunostained bands confirmed the TMT-MS detected decorin-decrease in lethal patients. Interestingly, different profiles of this proteoglycan were delineated in investigated samples. While *COL1A1* mutants presented only the 40 kDa immunoreactive band, *COL1A2* mutants showed different decorin proteoforms resolved into three different bands, at 40, 46, and 50 kDa. The decorin profile of fibroblast presenting mutations in *COL1A2* was similar to that of control cells. As protein-species from the same unique protein usually differ in chemical and physical properties, in structure, localization, and indeed in function, differential decorin post-translational modifications may have a role in disease, but further investigation will need to be performed.

### 2.3. SPARC Abundance Negatively Affects OI Outcome

The deleterious decrease of decorin in lethal patients seems acquiring a further negative relevance when associated to the over-presence of the collagen-binding matricellular glycoprotein SPARC that was detected in type II probands. SPARC, also known as osteonectin, was reported to influence ECM composition and structure, ECM:cell interaction and related induction and signaling [72,73]. The relevance of SPARC in OI was definitely proved by the identification of mutations responsible for recessive OI type XVII (the OI type number refers to the online Catalog of Human Genes and Genetic Disorders, OMIM) (# 616507) [74].

SPARC is proposed to contribute in collagen maturation and trafficking, and, by interfering with collagen-integrin (α_1_β_1_, α_2_β_2_, and α1_1_β_1_) engagement, to affect collagen fibrillogenesis and phagocytosis [75]. As osteonectin enhances TGF-β activity [73], its up-regulation may amplify the deleterious effects, we suggested above, of massive TGF-β release following ECM degradation, concurring to chronic inflammation during wound healing in OI patients.

### 2.4. Chondroitin Sulfate Proteoglycan 4: A Sprout Protein between Extra and Intracellular Compartment

Chondroitin sulfate proteoglycan 4 (CSPG4) is a highly glycosylated transmembrane glycosaminoglycan (GAGs) that binds collagens V and VI competitively with decorin [76] and that was found consistently upregulated in lethal OI patients. Since pericellular and extracellular proteoglycans control fibrillary collagen organization, morphogen gradients and their related signal transductions, as also described for decorin, CSPG4 is ubiquitously involved in tissue development and homeostasis. In fact, it interacts with ECM, ECM metalloproteases (MMPs), growth factors, integrins, and cytoskeletal anchoring proteins. CSPG4 is usually expressed in immature progenitor cells and it has been recently described controlling body axis organization in zebrafish gastrula [77]. In cancer cells, CSPG4 over-expression is retained enhancing local concentration and/or activation of MMPs, thus likely altering collagen type I degradation and consequent metastatic spreading [78]. Since this proteoglycan expression is regulated by inflammatory cytokines, such as TNF-α, TGF-β, and IL-1 [79], the excessive TGF-β release during bone remodeling may worsen ECM thickness, organization, and functionality by acting on CSPG4.

### 2.5. Tyrosine-Protein Kinase 7 (PTK7) Points to the Wnt Pathway as Modulator of OI Outcome

Along with decorin, the integral membrane protein inactive tyrosine-protein kinase 7 (PTK7) was associated to canonical and non-canonical Wnt signaling [80], as underlined by its “Wnt signaling pathway” and “Canonical Wnt signaling pathway” GO terms, both clustered in the *cell signaling* group. According to the MetaCore generated network (Figure 4), PTK7 is the only experimental protein acting on β-catenin, with this latter controlling several of the differentially expressed proteins we detected in type II and type III OI fibroblasts (Figure 4). PTK7 is retained to favor β-catenin stabilization and transcriptional activity [81]. Wnt signaling is a key process in osteoblastogenesis and in bone homeostasis control and its impairment causes several bone defects in OI, as demonstrated by Keupp and Pyott [82,83] that reported Wnt mutations as causative for the recessive OI type XV (MIM #615220). Altered expression of PTK7, by acting on Wnt signaling, may indeed represent a burden for OI cells.

PTK7 is also involved in proliferation, actin cytoskeleton reorganization, adhesion, migration, polarity, and apoptosis. In fact, the BP GO terms “cell adhesion” and “actin cytoskeleton reorganization” annotating PTK7 were included in the *cytoskeleton organization* group, and “ventricular septum development” and “coronary vasculature development” in the *differentiation and development* cluster. Indeed, the deregulation of this factor is also expected to have a role in OI aberrances related to cytoskeleton organization as described below.

### 2.6. Altered Cytoskeleton Modulates OI Phenotype

BP scatterplot central bubbles focalized attention on cytoskeleton organization (Figure 1) as a cellular feature that may compromise the capability of the probands to survive.

In addition to proteins already discussed, the MetaCore generated network evidenced a pivotal role acted by β-catenin in controlling several of the proteins of interest, which are involved in development/differentiation and cytoskeleton dynamics, (Figure 4). Among them, the **cysteine and glycine-rich protein 2** (CSRP2), which is a short LIM domain protein (21 kDa) localized in cytoplasm and nucleus, resulted more abundant in type II than in type III OI fibroblasts. Its increased expression was observed inducing smooth muscle differentiation [84] but also dedifferentiation in cancer cells [85,86]. Despite its function in cytoplasm is not clear, CSRP2 is involved in cytoskeleton remodeling by binding to F-actin [86]. The **Microtubule-associated protein 1A** (MAP1A) is another protein under the β-catenin control. MAP1A regulates the structure of microtubule assemblies and acts as a cytoskeleton linker protein interconnecting microtubules and actin filaments [87]. Despite its role in cytoskeleton dynamics is only partially clarified, MAP1A was described influencing intracellular transport and its overexpression seems to induce excessive stabilization of microtubules [87]. Consequently, the increased amount of MAP1A in lethal patients may concur to reduce cytoskeleton plasticity, which is crucial during cell migration, proliferation, polarization, organelle and vesicle trafficking, and indeed during differentiation and wound healing [10,61,88,89,90,91,92,93]. Β-catenin also controls **palladin**, a cytoskeleton associated protein, that we detected significantly increased in type II OI probands, even when compared to control subjects (Table 2). Palladin is involved in actin cytoskeleton and focal adhesion organization and stabilization [94,95] and, although its presence is required for actin microfilament organization, its over-presence may cause, as suggested for MAP1A, excessive rigidity of the cytoskeleton. The combined up-regulation of MAP1A and palladin may hence results in cytoskeleton affection by reducing its assembly and disassembly dynamism. Furthermore, β-catenin also controls the **protein S100-A4,** which we previously found down-regulated in type VII OI [10]. Here, we detected its down regulation in probands with lethal outcome (Table 2). S100A4 is a cytoskeleton modulating protein whose expression regulates osteoblast functions [96]. S100A4 is also a mediator of pro-inflammatory pathways and its defective presence may worsen wound healing processes [97]. Noteworthy, S100A4 induces MMP13 expression [98], that is one of the players of type I collagen degradation during wound repair [99]. Its defective presence in probands with the lethal outcome of the disorder may indeed determine defects in ECM degradation.

Finally, β-catenin regulates the expression of the integral membrane protein **neprilysin** (NEP), that is, among the 17 significant differences we detected, the protein with the lowest amount in lethal subjects [100]. Besides its control on local concentrations of small bioactive extracellular peptides by its extracellular zinc-metalloprotease activity, NEP is involved in membrane-domain and cytoskeleton organization and RhoA signaling by interacting, with its cytoplasmic N-terminal tail, with the ezrin/radixin/moesin (ERM) proteins [101,102]. Since this endopeptidase modulates the phosphatase and tensin homologue (PTEN) [103], which is the main negative regulator of the PI3K/Akt pathway [104], neprilysin may be even involved in cellular metabolism and osteoblast differentiation. Notably, Ruchon et al. [105] detected NEP on all bone-forming cells in mouse, where it resulted early expressed and with higher levels in newborns. The authors also proved neprilysin to cleave osteoactive substrates, i.e., osteostatin, osteogenic growth peptide (OGP), parathyroid hormone–related peptide 1-43 (PTHrP1-34), calcitonin, and α-calcitonin gene-related peptide (α-CGRP), and they supposed a NEP rule in ECM-integrity regulation by osteocytes. Moreover, neprilysin is also reported to interfere with focal adhesion kinase signaling by binding lyn kinase [106].

In conclusion, based on its numerous biological functions [107], neprilysin decreased presence may have serious effects in concentrations of extracellular bioactive-molecules, in membrane/cytoskeleton micro-domains organization, and related signal transduction. These may indeed result in defective differentiation and physiology of different cell types and organs, such as bone, brain, heart, lung, and kidney [107].

With a consistent increased presence in lethal patients we investigated (Table 2), the type VI intermediate filament (IF) protein **nestin** is the highest protein-difference identified by TMT analysis between OI type II and OI type III fibroblasts. According to its main associated BP GO terms (i.e., “positive regulation of neural precursor cell proliferation”, “central nervous system development”, and “positive regulation of intermediate filament depolymerization”), nestin is involved in *cell signaling*, *differentiation and development*, and *cytoskeleton and nucleoskeleton organization*. Nestin is expressed in a variety of stem cells and plays a key role in differentiation and development of neural and muscular systems [108]. It interferes in cytoskeleton architecture and dynamics mainly by modulating assembly and disassembly of other intermediate filaments (IFs) and, in particular, by promoting the disassembly of phosphorylated vimentin filaments [109,110]. Decorin decrease combined with nestin increase may hence lead to massive vimentin-intermediate filament disorganization and consequent cytoskeleton severe defects in OI lethal patients.

**Figure 6:**
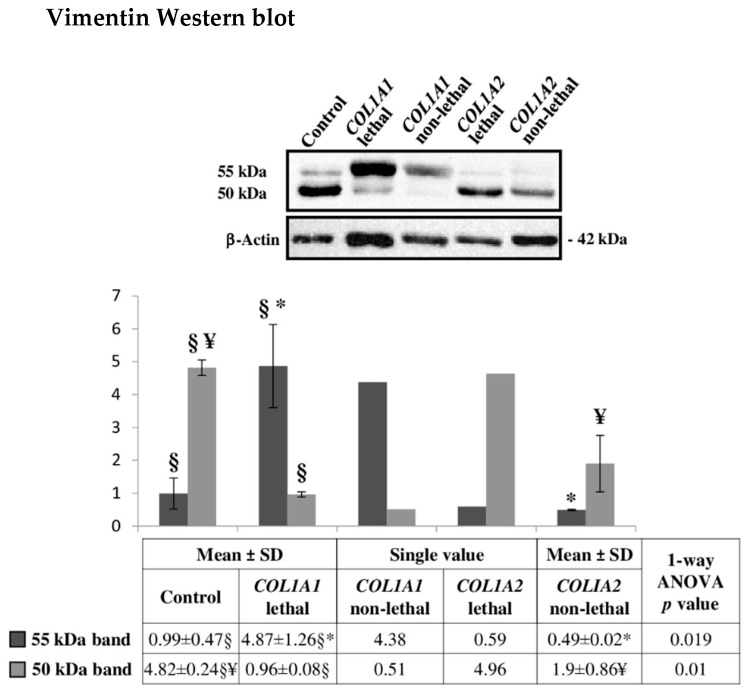
Representative vimentin Western blot on fibroblasts from dominant OI patients with lethal or non-lethal phenotype. The shown signals of immunostained bands from controls, *COL1A1* lethal and *COL1A2* non-lethal mutants are representative of the corresponding sample classes. Histograms visualize the mean of normalized relative-integrated-density ± SD values of controls (*n* = 3), *COL1A1* lethal (*n* = 2) and *COL1A2* non-lethal (*n* = 2) subjects, and normalized relative-integrated-density for *COL1A1* non-lethal (*n* = 1) and *COL1A2* lethal (*n* = 1) fibroblasts. Statistics was performed on normalized mean relative-integrated-density ± SD values, which were computed on values of Western blot signals from controls (*n* = 3), lethal *COL1A1* mutants (*n* = 2), and non-lethal *COL1A2* mutants (*n* = 2). §, ¥, and * symbols indicate the significance of abundance changes occurring between control and *COL1A1* lethal, control and *COLIA2* non-lethal, and *COL1A1* lethal and *COLIA2* non-lethal fibroblasts, respectively. The high SD is due to the wide individual biological heterogeneity of the analyzed samples.

Our analysis did not evidence differences in vimentin abundance between type II and type III OI fibroblast. Nonetheless, vimentin has a complex post-translational modification pattern [111] that may have escaped the TMT MS analysis. Thus, vimentin amount was analyzed by Western blot. Two immunoreactive bands, with MW between 50 and 55 kDa, were detected (Figure 6). Interestingly, vimentin bands revealed a characteristic trend in intensity, associated to the type I collagen mutated chain rather than to the outcome of the disease. Regardless of the severity, α1 (I) and α2 (I) mutants significantly differ in both vimentin bands (Figure 6). According to normalized relative integrated density values, lethal and survived α1 (I) mutants presented higher signal intensity in the 55 kDa band, while lethal and non-lethal α2 (I) mutants showed higher signals in the 50 kDa band. As discussed for decorin, although the overall concentration of an individual protein does not change, differential co- and post-translational modifications, resulting in a plethora of proteoforms, may modulate protein properties and functions.

**Figure 7:**
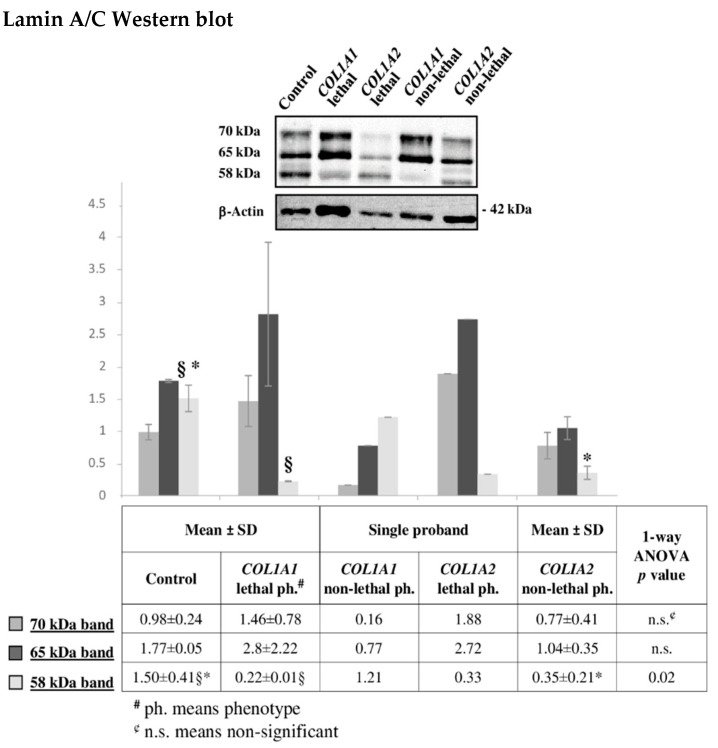
Representative lamin A/C Western blot on fibroblasts from dominant OI patients with lethal or non-lethal phenotype. The shown signals of immunostained bands from controls, *COL1A1* lethal and *COL1A2* non-lethal mutants are representative of the corresponding sample classes. Histograms visualize the mean of normalized relative-integrated-density ± SD values of controls (*n* = 3), *COL1A1* lethal (*n* = 2) and *COL1A2* non-lethal (*n* = 2) subjects, and normalized relative-integrated-density for *COL1A1* non-lethal (*n* = 1) and *COL1A2* lethal (*n* = 1) fibroblasts. Statistics was performed on normalized mean relative-integrated-density ± SD values, which were computed on values of Western blot signals from controls (*n* = 3), lethal *COL1A1* mutants (*n* = 2), and non-lethal *COL1A2* mutants (*n* = 2). § and * symbols indicate the significance of abundance changes occurring between control and *COL1A1* lethal, and control and *COL1A2* non-lethal phenotype, respectively. Significant differences were detected only for the 58 kDa band in the comparisons: control vs. *COL1A1* lethal phenotype and control vs. *COL1A2* non-lethal phenotype. The high SD is due to the wide individual biological heterogeneity of the analyzed samples. *COL1A1* mutant with lethal outcome presented a particular lamin A/C patter.

Besides vimentin, lamin A/C is another IF protein we previously described presenting defective abundance of different proteoforms in OI [10]. As for vimentin, TMT MS results did not evidenced differences in its presence between the lethal and the non-lethal outcomes. Nevertheless, we investigated lamin A/C pattern by Western blot (Figure 7). Three main immunoreactive bands were detected suggesting the presence of post-translational modifications. The lamin A/C pattern, observed in α1 (I) and α2 (I) mutants, highlighted proteoform defective occurrence among control cells and probands as well as between patients presenting mutations in α1 (I) or in α2 (I). The differences were related to the affected chain rather than to the severity of the disorder, with α2 (I) substitutions presenting a more similar pattern to control cells. However, the lethal α2 (I) substitution presented a distinctive pattern, when compared with the non-lethal α2 (I) mutations, by showing a signal decrease of the 70 kDa band and an increase of the 58 kDa one. However, more samples will be necessary to validate this observation.

Nonetheless, immunodetection confirmed lamin A/C dysregulation in OI patients evidently concurring to OI features. Lamin A and C are one of the major components of the nuclear lamina in differentiated cells and they perform a crucial role in transducing mechanical signals from the ECM, through the cytoskeleton, to the nucleus. In particular, they are fundamental in bone physiology. Lamin A and C controls bone formation, remodeling, and wound healing by orchestrating osteoblastogenesis and osteoclastogenesis in accordance with ECM biochemical and mechanical properties [112]. Furthermore, the lamin A/C defective occurrence we observed in dominant and recessive [10] OI may associate to the impaired transcription that was recently described in OI murine models [113].

## 3. Materials and Methods

### 3.1. Patients

Human fibroblasts from skin biopsy of OI patients, carrying mutations in *COL1A1* (*n* = 3) or in *COL1A2* (*n* = 3) genes and controls were obtained after informed consent and used at passage P8–10 (Table 1). The clinical description of the patients is reported in [2,114,115,116]. All cells from lethal OI cases were collected from fetuses or newborns, whereas the non-lethal OI samples were collected from babies. All patients were male with the exception of α 2(I)-G697C (Table 1). The controls cells were from individuals of pediatric age. The ethical approvals for the skin biopsies were obtained at the reference hospital centers, as reported in [2,114,115,116].

### 3.2. Tissue Culture and Lysate Preparation

Cells were grown at 37 °C in humidified atmosphere containing 5% CO2 and cultured in Dulbecco modified Eagle’s medium (D-MEM) (4.5 g/l glucose) (Lonza, Visp, Switzerland) supplemented with 10% (*v/v*) fetal bovine serum (FBS—Euroclone, Milan, Italy), 4 mM glutamine (Euroclone), 100 μg/mL penicillin and streptomycin (Euroclone). 2.5 × 10^4^ cells/cm^2^ were plated and cultured for 5 days with no media change. Then, cells were washed twice with cold phosphate-buffered saline (PBS) and scraped. The cell pellet was immediately frozen at −80 °C.

### 3.3. Sample Preparation for Tandem Mass Tag Labeling and LC-MS/MS

Cell pellets were suspended in RapiGest 0.1% (in TEAB 0.1 M, pH 8). Volumes were adjusted to 60 µL with RapiGest 0.1% (in TEAB 0.1 M, pH 8). Reduction of 20 ug of protein per sample was done with TCEP (tris(2-carboxyethyl)phosphine), final concentration of 50 mM, and samples reacted for 30 min at 60 °C. Alkylation was done with iodoacetamide, added to a final concentration of 15 mM, and samples incubated 60 min in the dark at room temperature. Trypsin was added (ratio of 1:25 (*w/w*)), and the digestion was performed overnight at 37 °C. Then, a 10-plex tandem mass tags (TMT) (Thermo Scientific, Rockford, IL, USA) labeling was performed according to manufacturer’s instructions. TMT reagent tags were dissolved in ACN, and each sample was incubated 60 min at room temperature with a specific tag. For TMT quenching, 8 µL of hydroxylamin 5% (*v/v*) were added, and incubated with the samples for 15 min. TMT-labeled samples were pooled. RapiGest was cleaved with TFA (pH < 2, incubated 45 min at 37 °C), centrifuged in a table centrifuge at 14,000 rpm for 10 min, supernatant was recovered and dried under vacuum. Sample are dissolved in 5% (*v/v*) ACN/0.1% (*v/v*) FA and desalted with C18 micro-spin columns (Thermo Scientific). Peptides were separated by off-gel electrophoresis, desalted, and solubilized in an appropriate amount of 5% (*v/v*) ACN / 0.1% (*v/v*) FA for mass spectrometry analysis.

For liquid chromatography-tandem mass spectrometry (LC-MS/MS), peptides were dissolved in 5% (*v/v*) ACN/0.1% (*v/v*) FA to a concentration of 0.25 µg/µL. Mass spectrometry experiments were performed on a Q Exactive Plus instrument from Thermo Scientific (San Jose, CA, USA) equipped with an Easy-nanoLC from Thermo. Peptides were trapped on 2 cm × 75 µm ID, 3 µm pre-column, and separated on an easy-spray column, 50 cm × 75 µm ID, PepMap C18, 2 µm (Thermo Scientific). The analytical separation was run for 60 min using a gradient of H_2_O/FA 99.9%/0.1% (*v/v*) (solvent A) and CH_3_CN/FA 99.9%/0.1% (*v/v*) (solvent B) at a flow rate of 300 nL/min. For MS survey scans, the OT resolution was set to 140,000 and the ion population was set to 3 × 106 with an m/z window from 350 to 2000. Twenty precursor ions were selected for higher-energy collisional dissociation (HCD) with a resolution of 35,000, an ion population set to 1 × 105 (isolation window of 0.5 m/z) and a normalized collision energy set to 30%.

### 3.4. Protein Identification and Quantification

Raw data were converted and peak lists were submitted to Easyprot for identification (Uniprot/SwissProt human database, 2014_10), and Isobar for quantification [117]. Carbamidomethylation of cysteines, TMT-sixplex amino terminus, TMT-sixplex lysine (for TMT labeled samples) were set as fixed modifications and methionine oxidation as variable modification. Two unique peptide sequences were the minimal required for protein identifications with a false discovery ratio (FDR) set as 1%. Quantification was achieved with IsoQuant, an open-source R package integrated into Easyprot. Isoquant performs the quantification on peptides not shared among different proteins. Statistics were performed according to Libra’s statistical implementation, a method adapted from the Trans-Proteomic-Pipeline (http://tools.proteomecenter.org/wiki/index.php? title = Software:Libra). After isotopic purity correction and abundance normalization, ratios were calculated as ratios of all peptides median. *p*-values were assessed by a Student’s *t*-test on averaged intensities of all peptides between both groups, to determine if the means of each group are significantly different.

Obtained statistically significant differences were further filtered for fold change and they were considered of interest with quantitative ratios above 1.5 or below 1/1.5.

### 3.5. Gene Ontology (GO) Clustering

“Biological process” (BP), “Molecular function” (MF), and “Cellular component” (CC) Gene Ontology (GO) terms, describing the differentially abundant proteins identified between lethal and non-lethal OI groups, were retrieved from UniProtKB (http://www.uniprot.org/; 2020_04 release: 188,244,498 UniProt/TrEMBL entries) and by applying QuickGO browser [118,119] (http://www.ebi.ac.uk/QuickGO/). Obtained GO-term lists were then processed by the REVIGO resource by setting a 0.7 cut-off value and *Homo sapiens* as referred database. REVIGO reduced GO term redundancy according to semantic similarity by applying the clustering/summarizing algorithm [120] (http://revigo.irb.hr/). The resulting sets of medium-length non-redundant GO-terms were then visualized through the scatterplot visualization option supported by the REVIGO server.

### 3.6. MetaCore Functional Analysis

The differentially expressed proteome dataset (*n* = 17 proteins) was further analyzed by the MetaCore™ resource (Clarivate Analytics, London, UK) for investigating protein functional enrichments and interconnections. The UniProtKB accession number (AN) list was imported into MetaCore and processed for functional enrichment by the “diseases by biomarkers” MetaCore ontology and by the “GO processes” public ontology by using the Functional Ontology Enrichment tool. The “diseases by biomarkers” enrichment analysis clusters proteins that are annotated as statistically significant biomarkers in characterized pathologies, and the “GO processes” analysis correlates experimental proteins with defined biochemical and molecular processes of biological systems.

The shortest path algorithm of the MetaCore Network Building tool software allowed visualizing the tight functional correlation existing among proteins of interest. It actually includes in the same net only experimental proteins that directly interact or that are functionally correlated by a further non-experimental protein, which acts as a molecular functional bridge. The resulting pathway maps were indeed prioritized, according to their statistical significance (*p* ≤ 0.001), and graphically visualized as nodes (proteins) and edges (interconnections among proteins).

### 3.7. Western Blotting

Protein extracts from all control and pathological fibroblasts were obtained in presence of the Laemmli buffer: 100 mM Tris–HCl pH 6.8, 2% (*w/v*) SDS, 20% (*v/v*) glycerol, 4% (*v/v*) β-mercaptoethanol, and heated at 95 °C for 5 min [121]. Twenty-five micrograms from each samples were separate by SDS-PAGE and subsequently transfer onto a nitrocellulose membrane (Hybond ECL—GE Healthcare, Billerica, MA, USA), as previously described [18]. Membranes were then blocked and differentially probed with a 1:1000 dilution of a mouse monoclonal anti-decorin antibody (Ab) (sc-73896) from Santa Cruz Biotechnology (San Jose, CA, USA); a 1:2000 dilution of a rabbit polyclonal anti-lamin A/C Ab (sc-20681) from Santa Cruz Biotechnology; and a 1:1000 dilution of a mouse monoclonal anti-vimentin Ab (sc-6260) from Santa Cruz Biotechnology. A 1:7000 dilution of polyclonal goat anti-rabbit IgG Ab (A0545) from Sigma-Aldrich (Saint Louis, MO, USA) and a dilution of 1:3000 of polyclonal goat anti-mouse IgG Ab (1706516) from Bio-Rad (Hercules, CA, USA) were used as secondary antibody.

Immunoreactive bands were visualized by chemiluminescence using ECL detection reagents (GE Healthcare) and chemiluminescent signals were captured by ChemiDocTM imaging system (Bio-Rad). Western blot images were then analyzed with the ImageQuant v. 3.0 software (Molecular Dynamics, Sunnyvale, CA, USA) and the ImageMaster 2D Platinum v. 6.0 software (GE Healthcare). B-actin immunoblotting ensured equal loading of samples in each lane. Mouse monoclonal anti-β-actin (A5441) from Sigma-Aldrich, 1:40,000 diluted was used as the primary antibody.

Statistic differences of means of normalized relative-integrated-density values (computed for individual immunoreactive bands) between groups were evaluated by one-way ANOVA. A pairwise comparison of experimental groups was also performed by the Tukey’s post-hoc multiple comparison test using the Exel Template interSTAT-a 2.0.

## 4. Conclusions

By applying a TMT LC-MS/MS approach and bioinformatic tools for comparative proteomic profiling, our findings corroborate the significant role played by defective regulation of intracellular and extracellular proteins in OI severity. With the limit imposed by the small number of analyzed patients and the consequent preliminary value of the obtained results, the comparative protein-abundance profiling and the predictive functional analysis allowed the identification of potential biomarkers (Table 2), not previously associated to OI probands’ lethality, whose expression pattern may contribute to mutants’ failure in balancing aberrances related to the disease.

Our study points to the hypothesis that OI molecular and biochemical impairment depends on a “domino reaction” where the potential biomarkers we delineated may act on and differentially affect, in reason of their differential presence, ECM dynamics (e.g., decorin, fibrillin-1, chondroitin sulfate proteoglycan 4 (CSPG4), and neprilysin), membrane micro-domain and cytoskeleton organization (e.g., nestin, paladin, decorin, inactive tyrosine-protein kinase 7 (PTK7), Microtubule-associated protein 1A (MAP1A), protein S100A4, and neprilysin), cell adhesion (e.g., PTK7), protein and vesicle trafficking (e.g., ADP-ribosylation factor 4 (ARF4), syntaxin-7, SPARC, and MAP1A), and nucleoskeleton arrangement, signal transduction, and gene expression (e.g., fibrillin-1, decorin, CSPG4, SPARC, PTK7, S100A4, neprilysin, and nestin).

We are aware that further analyses are necessary to confirm the obtained data, nevertheless, our work suggests potential crucial players in the genotype-phenotype relationship. Despite few, identified differences are in fact highly significant in the OI pathological context. They represent key factors in controlling, taking part, and/or modulating molecular processes whose affection could contribute to OI phenotypic traits.

Notably, we pointed out that common affected pathways and similar molecular defects, secondary to specific and distinctive gene mutations, characterize different heritable disorders [10,122,123], thus providing a sort of signature of potential phenotype modulators. Defining these critical effectors is relevant both to understand the disease pathophysiology and to identify novel and more precise therapeutic targets.

## Figures and Tables

**Table 1:** Patients’ fibroblasts analyzed in the study.

Gene	Protein Defect ^$^	OI Type ^#^	Gender	Age ^§^	PPERK/PERK	LC3II+ Chloroquine	CleavedCasp3
COL1A1	α1(I)-G226S	III	M	8 m	↑	↑	↑
COL1A1	α 1(I)-G478S	II	M	0 m	↑	↑	↑
COL1A1	α 1(I)-G994D	II	M	f	↑	↑	↑
COL1A2	α 2(I)-G640C	II/III *	M	6 m	~	~	↑
COL1A2	α 2(I)-G697C	II	F	1 m	↑	↑	↑
COL1A2	α 2(I)-G745C	III	M	3 y	↑	~	↑

* The patients died at 6 m for cardiorespiratory failure; ^$^ amino acid number considering the initial methionine codon as amino acid; ^#^ OI type based on Sillence’s classification; ^§^ m: month; y: year; f: fetus.

**Table 2:** Tandem Mass Tag-Mass Spectrometry (TMT MS) identified differentially expressed proteins occurring between primary fibroblast cell lines from type II and type III osteogenesis imperfecta (OI). The table also listed ratio values of the Lethal patients vs. Controls (CRTL) and Survived patients vs. CTRL comparisons relatively to the identified proteins of interest in Lethal vs. Survived.

Swiss-Prot Accession Number	Swiss-Prot Identifier	Protein Description	Dead p. vs. CTRL	Dead p. vs. Survived p. ^¥^	Survived p. vs. CTRL
P08473	NEP_HUMAN	Neprilysin	0.55	0.50	NS
P26447	S10A4_HUMAN	Protein S100-A4	0.26	0.63	0.42
P21589	5NTD_HUMAN	5’-nucleotidase	0.40	0.64	0.69
P07585	PGS2_HUMAN	Decorin	0.27	0.64	0.43
P68431	H31_HUMAN	Histone H3.1	0.57	0.66	NS
P18085	ARF4_HUMAN	ADP-ribosylation factor 4	NS	0.66	NS
Q8WX93	PALLD_HUMAN	Palladin	2.06	1.51	1.35
P35555	FBN1_HUMAN	Fibrillin-1	NS	1.58	NS
O15400	STX7_HUMAN	Syntaxin-7	1.52	1.62	NS
P05091	ALDH2_HUMAN	Aldehyde dehydrogenase, mitochondrial	NS	1.63	NS
P78559	MAP1A_HUMAN	Microtubule-associated protein 1A	NS	1.68	NS
Q13308	PTK7_HUMAN	Inactive tyrosine-protein kinase 7	1.5	1.68	NS
P09486	SPRC_HUMAN	SPARC	2.91	1.69	1.66
P11216	PYGB_HUMAN	Glycogen phosphorylase, brain form	NS	1.70	0.6
Q6UVK1	CSPG4_HUMAN	Chondroitin sulfate proteoglycan 4	1.98	1.80	NS
Q16527	CSRP2_HUMAN	Cysteine and glycine-rich protein 2	2.44	1.88	NS
P48681	NEST_HUMAN	Nestin	2.11	2.33	NS

^¥^ All the obtained ratios are supported by high statistics with *p* ≤ 0.001. In the Lethal vs. CRTL and Survived vs. CTRL columns, non-significant ratio values (0.5 ≤ ratio ≤ 1.5 and/or *p* ≥ 0.001) are indicated by NS.

## Data Availability

Data is contained within the article or supplementary material.

## Supplementary Materials

The following are available online at https://www.mdpi.com/1422-0067/22/1/429/s1, Figure S1: REVIGO scatter plot of summarized GO Biological Process terms (Figure S1A); REVIGO scatter plot of summarized GO Molecular Function terms (Figure S1B); REVIGO scatter plot of summarized GO Cellular Component terms (Figure S1C).
