# Supplementary figures and images for: Intracellular and Extracellular Markers of Lethality in Osteogenesis Imperfecta: A Quantitative Proteomic Approach
Source: Int J Mol Sci. 2021 Jan 4;22(1):429. doi: 10.3390/ijms22010429 (PMC7795927; doi:10.3390/ijms22010429)

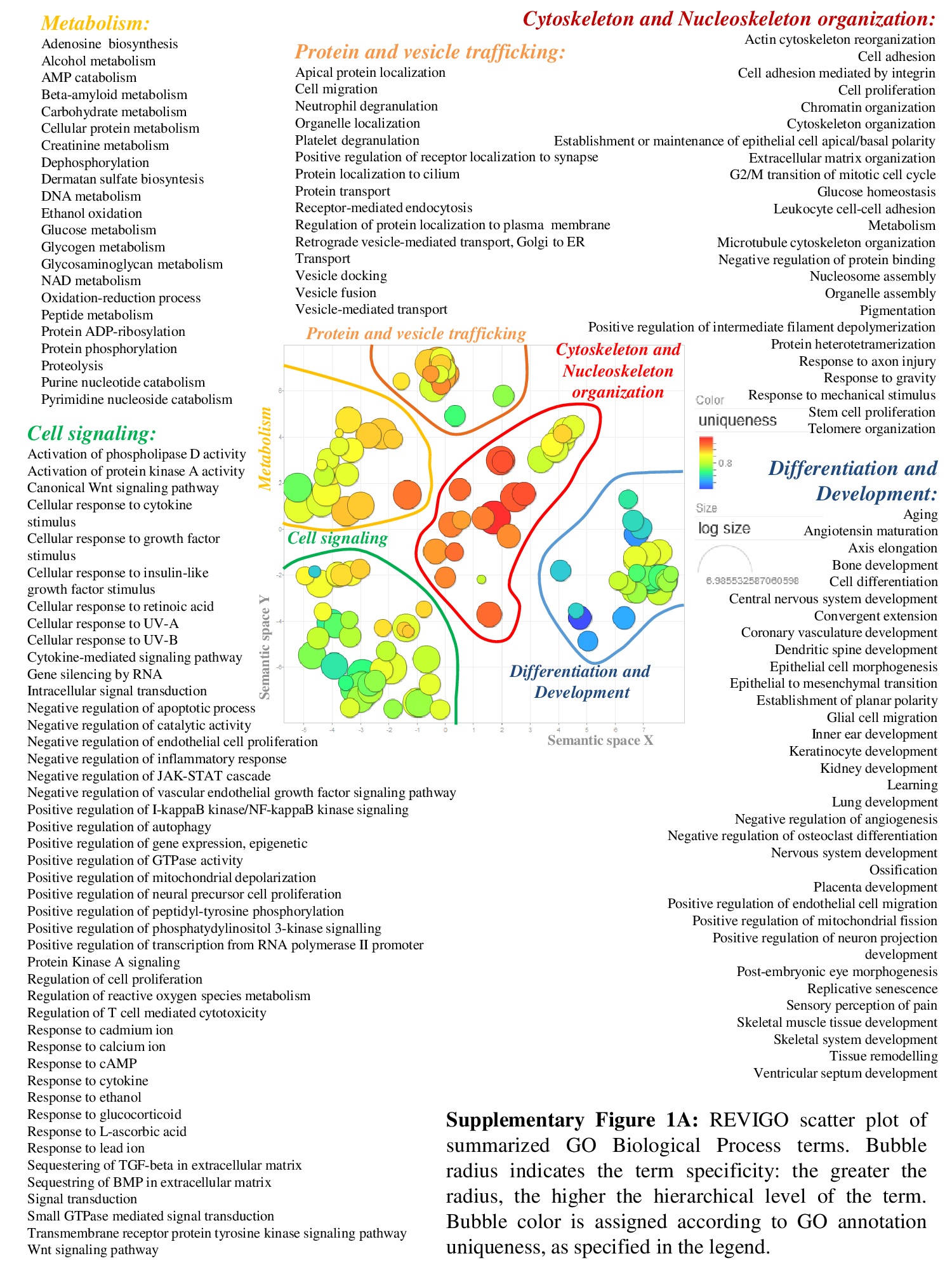

Supplement: Supplementary file 1 [file ijms-22-00429-s001.zip › ijms-1041346-Supplementary Materiala/Supplementary Figure S1A.jpeg]

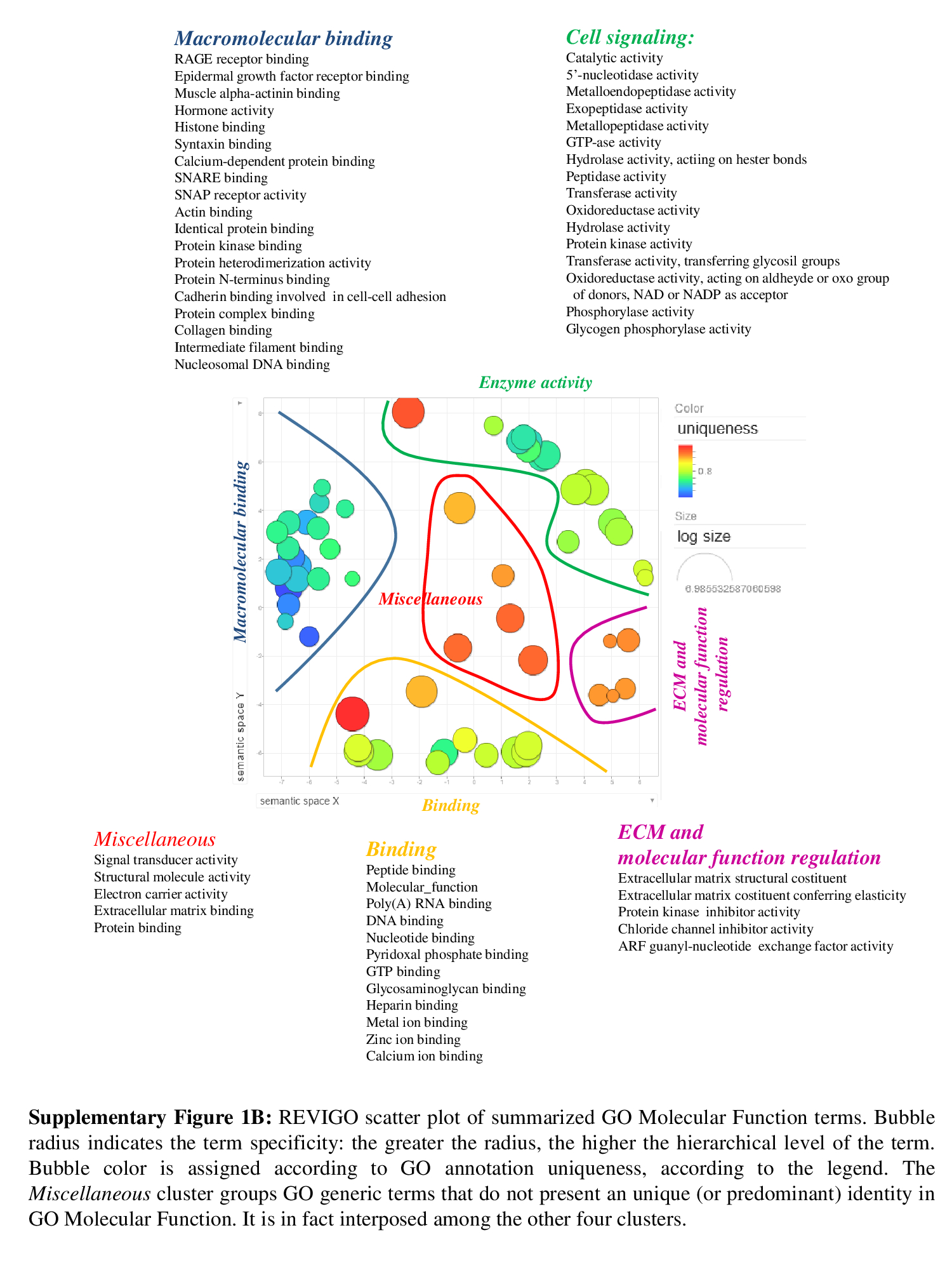

Supplement: Supplementary file 1 [file ijms-22-00429-s001.zip › ijms-1041346-Supplementary Materiala/Supplementary Figure S1B.jpeg]

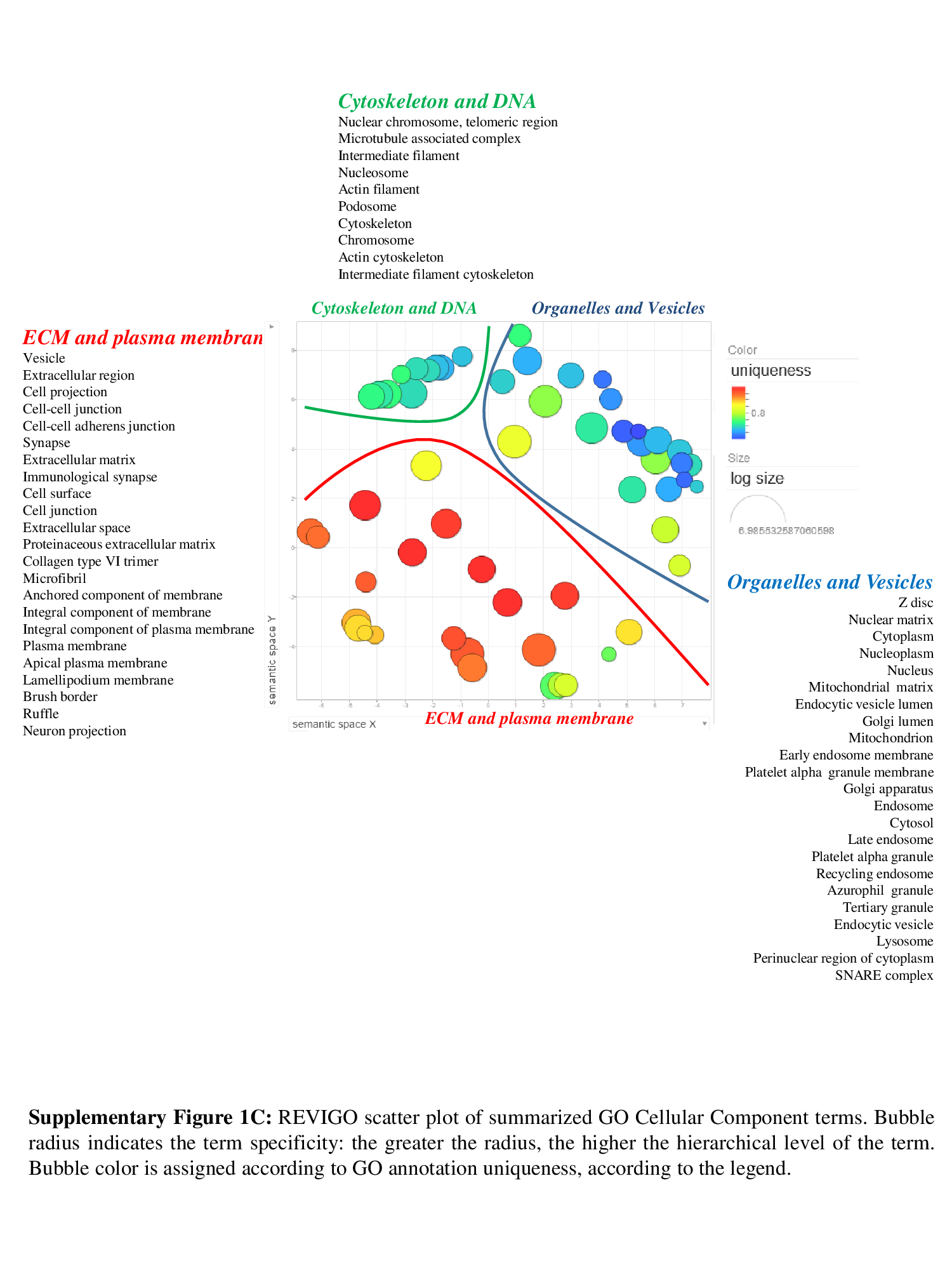

Supplement: Supplementary file 1 [file ijms-22-00429-s001.zip › ijms-1041346-Supplementary Materiala/Supplementary Figure S1C.jpeg]
